# Anti-Differentiation Effect of Oncogenic Met Receptor in Terminally-Differentiated Myotubes

**DOI:** 10.3390/biomedicines3010124

**Published:** 2015-02-12

**Authors:** Valentina Sala, Simona Gallo, Stefano Gatti, Elisa Vigna, Antonio Ponzetto, Tiziana Crepaldi

**Affiliations:** 1 Department of Oncology, University of Turin, Corso Massimo D’Azeglio 52, 10126 Turin, Italy; E-Mails: valentina.sala@unito.it (V.S.); simona.gallo@unito.it (Si.G.); stefano.gatti@unito.it (St.G.); 2 Department of Medical Sciences, University of Turin, Corso Dogliotti 14, 10126 Turin, Italy; E-Mail: antonio.ponzetto@unito.it; 3 Department of Oncology, Candiolo Cancer Institute—Fondazione del Piemonte per l'Oncologia (FPO) Istituto di Ricovero e Cura a Carattere Scientifico (IRCCS), Strada Provinciale 142, Km 3.95, 10060 Candiolo, Italy; E-Mail: elisa.vigna@ircc.it

**Keywords:** HGF, Met receptor, myogenic stem cells, skeletal muscle, differentiation, proteasome, Erk1,2 MAPK

## Abstract

Activation of the hepatocyte growth factor/Met receptor is involved in muscle regeneration, through promotion of proliferation and inhibition of differentiation in myogenic stem cells (MSCs). We previously described that the specific expression of an oncogenic version of the Met receptor (Tpr–Met) in terminally-differentiated skeletal muscle causes muscle wasting *in vivo*. Here, we induced Tpr–Met in differentiated myotube cultures derived from the transgenic mouse. These cultures showed a reduced protein level of myosin heavy chain (MyHC), increased phosphorylation of Erk1,2 MAPK, the formation of giant sacs of myonuclei and the collapse of elongated myotubes. Treatment of the cultures with an inhibitor of the MAPK kinase pathway or with an inhibitor of the proteasome increased the expression levels of MyHC. In addition, the inhibition of the MAPK kinase pathway prevented the formation of myosacs and myotube collapse. Finally, we showed that induction of Tpr–Met in primary myotubes was unable to produce endoreplication in their nuclei. In conclusion, our data indicate that multinucleated, fused myotubes may be forced to disassemble their contractile apparatus by the Tpr–Met oncogenic factor, but they resist the stimulus toward the reactivation of the cell cycle.

## 1. Introduction

Skeletal muscle regeneration relies on proliferation, differentiation and self-renewal of myogenic stem cells (MSCs). In healthy skeletal muscle, MSCs remain in a quiescent state. Injury activates MSCs, which exit from the G_0_ status and enter the cell cycle. After a proliferative phase, MSCs exit the cell cycle, start terminal differentiation through upregulation of muscle-specific genes, such as myosin heavy chain (*MyHC*), and fuse with each other and with adjacent fibers to form multinucleated myotubes [1]. A number of factors have been identified as major actors in inducing the activation and proliferation of MSCs, including hepatocyte growth factor (HGF) and its Met tyrosine kinase receptor, which is expressed by both quiescent and activated MSCs and subsequently downregulated in mature myofibers [2,3,4,5]. Release of HGF ligand from injured muscle activates the Met receptor on MSCs, induces their activation and increases their proliferation and migration to the site of injury. Recently, it has been suggested that the quiescent state of MSCs is more complex than previously thought, being constituted by two phases (G_0_ and G_Alert_) characterized by distinct proliferation and differentiation kinetics [6]. Interestingly, HGF is one of the factors capable of inducing the alert state of MSCs after injury. Some activated MSCs can reenter mitotic quiescence and maintain the so-called self-renewed myoblast population required to fuel the muscle regeneration process. It has been shown that high concentrations of HGF can drive MSCs back into the quiescent stage by inducing myostatin expression and limiting MSCs proliferation [7]. Thus, HGF seems to have double and opposite effects on MSCs. Moreover, HGF has been shown to be effective in inhibiting myogenic differentiation of C2C12 [8] and satellite cells (SCs) [4,9] *in vitro*. Consistently, injection of HGF in injured muscle *in vivo* blocks the differentiation of primary muscle cells [10].

To investigate whether HGF/Met signaling could give an adequate stimulus to counteract the differentiation program in terminally-differentiated myotubes, we analyzed the effects of a constitutively-activated form of Met receptor (Tpr–Met) in primary myotube cultures. The Tpr–Met oncogene, generated following chromosomal translocation, encodes a chimeric protein with constitutive tyrosine kinase activity, produced by the fusion of the leucine zipper domain of TPR with the tyrosine kinase domain of the Met receptor [11]. This hybrid Tpr–Met protein is able to cause a block of muscle differentiation in C2C12 myoblast cells [8]. In this work, we expressed Tpr–Met under control of the muscle creatine kinase (MCK) promoter, which is specifically induced in terminally-differentiated skeletal muscle. Our results suggest that constitutively-activated Tpr–Met signaling in myotubes downregulates the MyHC muscle differentiation marker. Forced constitutive Tpr–Met signaling elicits the formation of aberrant myotubes with aggregated nuclei (myosacs). Pharmacological inhibition of either the Erk1,2 MAPK pathway or proteasome-dependent degradation attenuates the Tpr–Met anti-differentiation effect. Finally, we show that activated Tpr–Met is not able to stimulate the nuclei of myosacs to re-enter the cell cycle and proceed into the S phase. In conclusion, the activity of Tpr–Met is able to oppose terminal differentiation, while it is not sufficient to reactivate the cell cycle in myotubes. These results suggest that the forced hyperactivation of Met signaling could be exploited in the development of a therapeutic approach for regenerative medicine. In fact, in the future, the constitutive Tpr–Met activity could be used for the reversal of the differentiation program of mammalian muscle in combination with factors inducing cell cycle reentry.

## 2. Results

### 2.1. Terminally Differentiated Myotubes from Tpr–Met Mice form Gigantic Myosacs and Collapse

**Figure 1:**
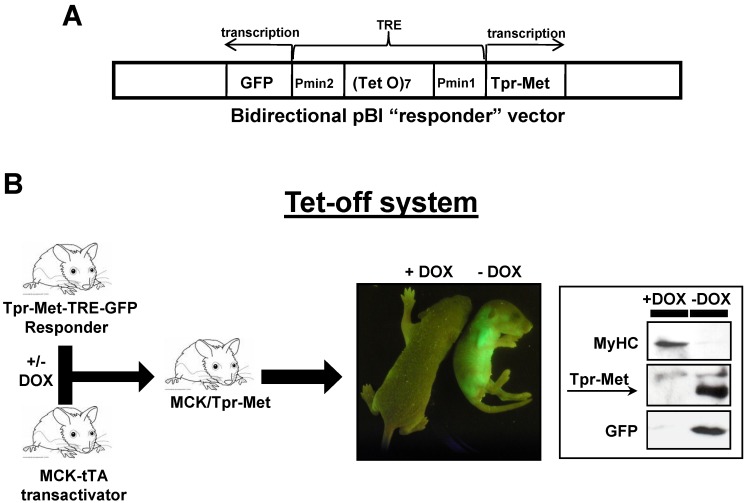
Inducible Tpr–Met–TRE–GFP/MCK–tTA mouse model. (**A**) Schematic representation of Tpr–Met–TRE–GFP transgene in the bidirectional pBI “responder” vector; (**B**) breeding strategy to generate bitransgenic mice (MCK/Tpr–Met), conceived and raised either in the presence or in the absence of doxycycline (DOX). Tpr–Met+/GFP+ pups (−DOX, middle panel) are smaller than controls (+DOX, middle panel). In the western blot (WB) of skeletal muscle, low levels of MyHC are shown in concomitance with induced expression of Tpr–Met and GFP proteins (right panel).

We previously generated a Tpr–Met–TRE–GFP transgenic responder mouse [12], in which the Tpr–Met and the *GFP* reporter genes are under control of a tetracycline responsive element (TRE) composed of bidirectional artificial “minimal” promoters (Pmin1 and Pmin2) carrying multiple operator sites (TetO)_7_ responsive to tetracycline (tc) (Figure 1A).

**Figure 2:**
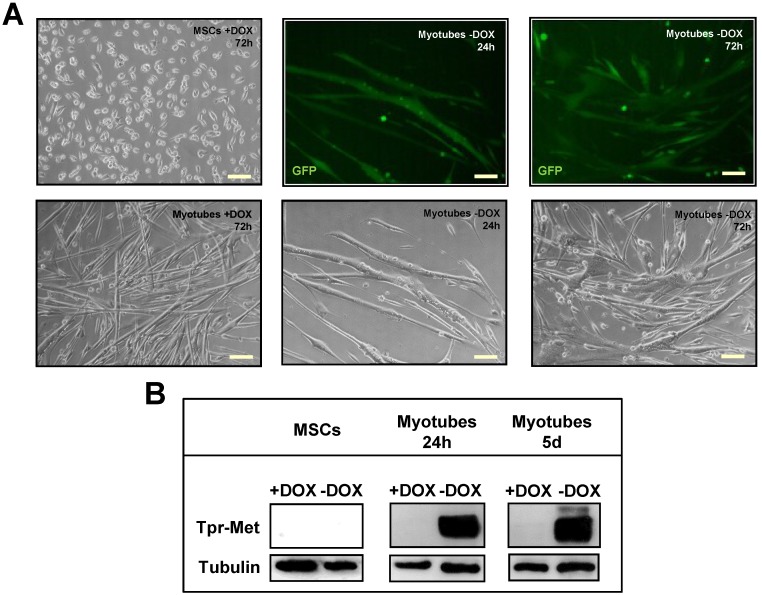

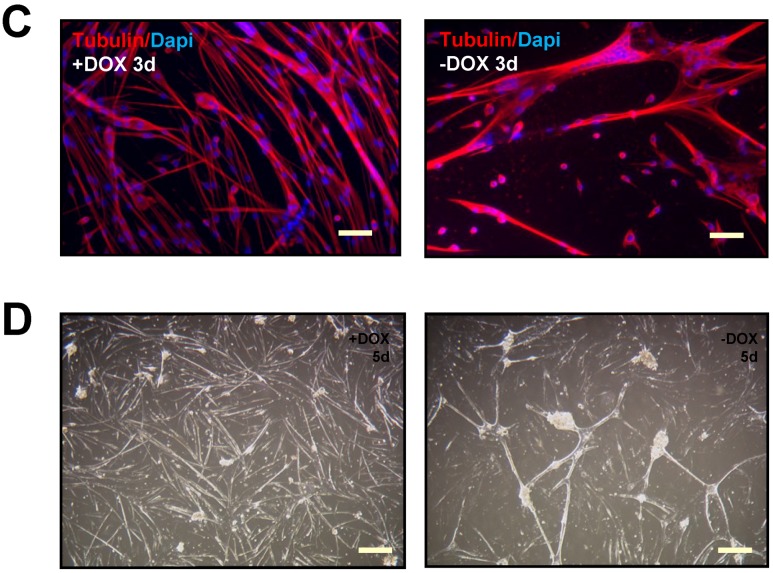
Characterization of proliferating myogenic stem cells (MSCs) and differentiated myotubes from bitransgenic mice. (**A**) Left panels: representative optical images of proliferating MSCs (upper) and differentiated myotubes (bottom) grown for 72 h in uninduced conditions (+DOX); Middle and right panels: representative GFP immunofluorescence (upper) and optical (bottom) images of differentiated myotubes cultured for 24 h (middle) or 72 h (right) in induced conditions (−DOX); (**B**) Tpr–Met protein analysis in western blot of MSCs and myotubes at 24 h or five days of differentiation, cultured in the presence or absence of DOX; (**C**) Immunofluorescence staining of α-tubulin (red) and DAPI (blue) in uninduced (left) and induced (right) myotubes after 72 h incubation in DM; (**D**) Optical images of uninduced (left) and induced myotubes (right) after incubation in DM for five days. Scale bars: (**A**,**C**) 100 μm; (**D**) 200 µm.

Expression of the transgene requires the presence of a tetracycline-regulatable transcription factor (tTA), which specifically transactivates the TRE. In this Tet-off system, adding the tc-like compound DOX to the system reversibly inhibits binding of tTA to the promoter and blocks gene expression [13]. The Tpr–Met–TRE–GFP responder mouse has then been crossed with a mouse line carrying the tTA transactivator (Tet-off) under control of the MCK promoter [14] (Figure 1B). The Tpr–Met–TRE–GFP/MCK–tTA bitransgenic mice express the “responder gene” specifically in skeletal muscle in the absence of DOX (Figure 1B). Only −DOX pups show green fluorescent muscles when examined under the appropriate light box and express Tpr–Met and GFP proteins in their skeletal muscles, as indicated by western blot (Figure 1B). These pups exhibit a smaller size when compared to DOX-treated controls, because of the severe reduction of the skeletal muscle mass and MyHC sarcomeric protein (Figure 1B). Mouse myogenic cultures were obtained through enzymatic dissociation from hindlimb muscles of bitransgenic animals kept in DOX from conception to repress transgene expression. Myogenic cultures yielded proliferating MSCs with a typical round morphology when cultured at low density in mitogen-rich growth medium (GM, Figure 2A, upper left panel). When MSCs were grown at high density and shifted into differentiation medium (DM), they fused to form multinucleated myotubes (Figure 2A, lower left panel). To verify the efficacy of the Tet-off system in culture, proliferating and differentiated myogenic cultures were grown in the presence or absence of DOX. When grown in the absence of DOX, MSCs did not express Tpr–Met protein (Figure 2B), while differentiated myotubes expressed both GFP (Figure 2A, middle and right upper panels) and Tpr–Met (Figure 2B, myotubes −DOX), starting at 24 h through five days from DM shift. These data indicate that the MCK promoter is silent in proliferating MSCs and is specifically activated only upon myotube fusion. Treatment with DOX kept the Tpr–Met expression off in myotubes (Figure 2B, myotubes +DOX). Altogether, these data indicate that differentiated myotubes express Tpr–Met in a doxycycline-regulatable manner.

When grown in DM, uninduced (+DOX) MSCs elongated, aligned and fused to form very long and thin multinucleated myotubes (Figure 2A, lower left panel, and Figure 2C, left panel). In contrast, terminally-differentiated myotubes induced to express the Tpr–Met transgene in the absence of DOX showed irregular fusion and formed GFP-positive X–Y shaped structures at 24 h from DM shift (Figure 2A, middle panels). After 72 h of incubation in DM, Tpr–Met expressing myotubes showed enhanced fusion, leading to gigantic structures with aggregated nuclei (Figure 2A,C right panels). After five days of culture in DM, Tpr–Met+ myotubes collapsed (Figure 2D).

### 2.2. Induction of Tpr–Met in Myotubes Downregulates MyHC through Erk1,2 MAPK Activation and Proteasome-Dependent Degradation

**Figure 3:**
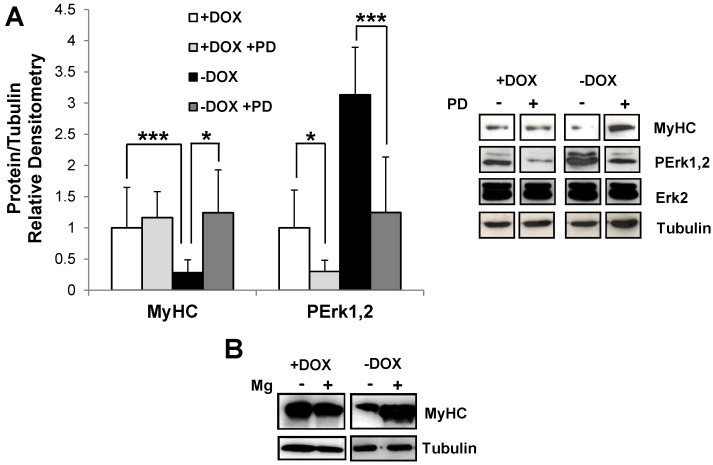

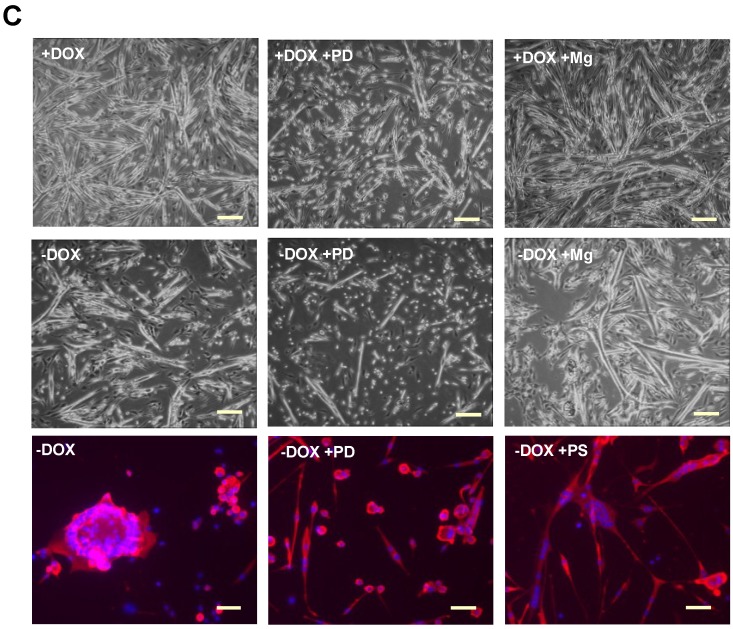
Changes of MyHC content and morphology in myotubes after treatment with MAPK pathway and proteasome inhibitors. (**A**) MyHC and phospho-Erk1,2 were analyzed and quantified by western blot of the lysates from uninduced (+DOX, white and light grey columns) and induced (−DOX, black and dark grey columns) myotubes cultured in the absence (white and black columns) or in the presence (light and dark grey columns) of the PD098059 inhibitor (PD) of the MAPK pathway. Values are the mean ± SD of three independent experiments and are expressed as the fold relative to the +DOX control (white column). Representative images of MyHC, PErk1,2 and total Erk2 are shown on the right. Tubulin was used as the loading control. For the MyHC experiment, a *t*-test was evaluated comparing +DOX *vs.* −DOX and −DOX *vs.* −DOX + PD samples. The *t*-test of PErk1,2 WB was calculated between +DOX *vs.* +DOX + PD and between −DOX *vs.* −DOX + PD. * *p* < 0.05, *** *p* < 0.005; (**B**) Analysis of MyHC in the western blot of the lysates from uninduced (+DOX) and induced (−DOX) myotubes, grown in the absence or in the presence of the Mg132 proteasome inhibitor (Mg); (**C**) Differentiated myotubes with or without induction of Tpr–Met were either untreated (left panels), treated with PD098059 (+PD, middle panels), or treated with inhibitors of the proteasome: Mg132 (+Mg, upper and middle right panels) and PS-341 (+PS, lower right panel). The upper and middle panels are optical images of myotubes cultured for three days in DM. The bottom panels are immunofluorescence images of myotubes cultured for five days in DM. Red, MyHC; blue, DAPI. Scale bars: 500 µm (upper and middle panels) and 100 μm (bottom panels).

Disassembly and collapse of Tpr–Met-positive myotubes suggested that degradation of muscle-specific cytoskeletal proteins occurred. We thus examined the quantitative levels of the muscle structural protein MyHC. MSCs were differentiated in DM for three days with and without DOX, and total protein extracts were probed by anti-myosin antibody (MF20) in the western blot. Tpr–Met-expressing myotubes (−DOX) showed a significantly reduced MyHC protein level, as compared with cultures grown in the presence of DOX (Figure 3A).

Since Tpr–Met is a powerful activator of the Ras/Raf/MAPK pathway [15], we investigated the activation state of Erk1,2 MAPK in uninduced (+DOX) and induced (−DOX) myotube cultures by western blot. Levels of phospho-Erk1,2 were found to be increased in myotubes with Tpr–Met induction (−DOX, Figure 3A). To assess the role of the MAPK pathway in MyHC downregulation, we used MAPK kinase MEK1 inhibitor PD098059 drug. The phosphorylation level of Erk1,2 MAPK was significantly reduced by the specific inhibitor in both conditions (Figure 3A). In parallel, we found that MyHC levels were increased by inhibitor treatment in induced cultures (−DOX), as compared with the uninduced control (+DOX, Figure 3A). Next, we asked whether MyHC downregulation was due to protein degradation via the proteasome. Treatment of Tpr–Met-induced myotubes with the proteasome inhibitor Mg132 markedly increased MyHC protein levels, while myotubes differentiated in uninduced conditions (+DOX) were unaffected by Mg132 inhibitor treatment (Figure 3B). Finally, we analyzed whether treatment with PD098059 inhibitor or with proteasome inhibitors (Mg132 and PS-341) could rescue the normal fusion and myotube integrity of Tpr–Met-induced cultures. MSC cultures were grown for 48 h after shifting to DM and then treated for a further 48 h with either inhibitors. As shown in Figure 3C (left and middle panels), both the formation of myosacs from Tpr–Met+ myotubes and their collapse were prevented by MAPK inhibitor. Exposure to PD098059 resulted in the shortening of myotubes also in uninduced cultures (Figure 3C, middle panels), indicating that Erk1,2 activity is required for myogenic differentiation to enhance the fusion of multinucleated myotubes. In contrast, treatment with proteasome inhibitors was not able to rescue the elongated phenotype, while it prevented the collapse of Tpr–Met+ myotubes (Figure 3C, right panels). Altogether, these results indicate an association between Tpr–Met-induced myotube collapse and protein degradation and suggest that the observed myotube rupture is likely due to the degradation of contractile proteins forming myofibrils.

### 2.3. Induction of Tpr–Met Fails to Elicit DNA Endoreplication in Myotubes

**Figure 4:**
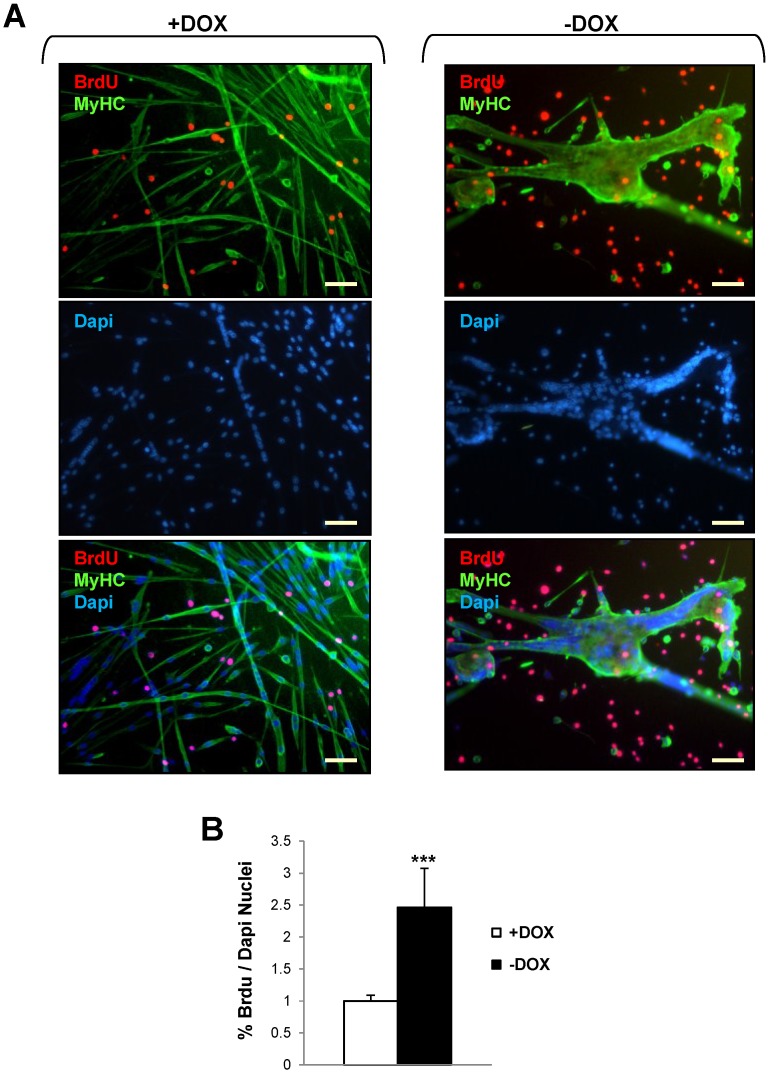
Induction of Tpr–Met fails to elicit BrdU incorporation in myotubes. Uninduced (+DOX, left panels) and induced (−DOX, right panels) myotube cultures grown in DM for two days were stimulated with FBS for a further two days and then incubated with 10 mM BrdU for 24 h. (**A**) Representative images of immunofluorescence. Upper panels: staining with anti-MyHC (green) and anti-BrdU (red) antibodies; Middle panels: total nuclei stained with DAPI (blue); Lower panels: triple overlay with MyHC, BrdU and DAPI. Scale bar: 100 μm; (**B**) The percentage of BrdU-positive nuclei with respect to the total amount of nuclei was counted. Values are the mean ± SD of 12 fields and are expressed as the fold relative to the +DOX control. The *t*-test was evaluated comparing −DOX *vs.* +DOX samples. *** *p* < 0.005.

To investigate whether the expression of Tpr–Met oncogene in multinucleated myotubes could restore their ability to synthesize DNA and force their re-entry into the cell cycle, we performed BrdU labeling experiments in uninduced (+DOX) and Tpr–Met induced (−DOX) differentiated myotubes (Figure 4). After three days in DM, myotubes were cultured for a further two days in DM or DM + 10% FBS and incubated with BrdU for a further 24 h to monitor cells undergoing the S phase. The double-immunofluorescence assay for BrdU and MF20 (Figure 4A) showed that thin myotubes of uninduced (+DOX) cultures and gigantic myosacs of Tpr–Met-induced (−DOX) cultures did not incorporate BrdU into their nuclei, indicating that the large majority of nuclei in the myotubes were not driven into the cell cycle. On the other hand, we observed an increased number of isolated mononucleated cells characterized by BrdU incorporation in Tpr–Met+ cultures, compared with uninduced cultures (Figure 4A,B).

## 3. Discussion

Until now, much of the work on the role of HGF/Met in muscle has been concentrated on activated SCs [5,9]. Upon release from injured myofibers, HGF binds and stimulates the Met receptor, which is necessary to induce SCs to leave the quiescent state and enter the cell cycle [6,10]. Terminal differentiation of newly-formed myotubes requires the irreversible withdrawal from the cell cycle, coupled to the upregulation of muscle-specific genes [1]. In line with this idea, by promoting the cell cycle in myoblasts, HGF inhibits their differentiation [4,8] and reduces the number or size of regenerating fibers *in vivo* [10]. In this work, we investigated whether forced hyperactivation of Met signaling in differentiated skeletal muscle could induce the reversal of the differentiation and re-entry of myotubes into cell cycle.

We found that the expression of the Tpr–Met oncogene, the constitutively activated form of the Met receptor, in terminally-differentiated myotubes *in vitro*, promotes MyHC protein degradation through a proteasome-dependent pathway. Proteolytic degradation of muscle proteins may be significant in view of the reactivation of the cell cycle, since the contractile system represents a serious physical barrier for chromosome segregation and cytokinesis. Thus, a non-dividing cell, like the myofiber, must degrade proteins characteristic of the terminally-differentiated state, in order to specify itself to the identity of a local progenitor cell. Furthermore, degradation of already existing proteins must occur in a reprogrammed cell, in which the protein synthesis profile drastically changes. Myogenic cultures expressing Tpr–Met show strong stimulation of Erk1,2 MAPK signaling, concomitantly with downregulation of the MyHC protein. This is consistent with previous works showing that stimulation of the Ras/Raf/MEK/MAPK pathway is able to promote muscle proteolysis in C. elegans [16,17]. Accordingly, Tpr–Met in fetal and adult muscle leads to muscle wasting *in vivo* [12]. Interestingly, the proteolysis-inducing factor, a cytokine produced by cachexia-inducing tumors, is able to activate the ubiquitin-proteasome proteolytic pathway through Erk1,2 MAPK [18].

In Tpr–Met+ differentiating myogenic cultures, prior to myotube collapse, we observed numerous abnormally-fused myotubes. Fusion of myoblasts progressed to gigantic structures containing big aggregations of nuclei, which resembled myosacs, obtained through the overexpression of transcription factors that regulate myoblast fusion [19,20]. The formation of the myosacs was prevented by treatment with the PD098059 inhibitor, while it remained unaffected by treatment with the proteasome inhibitor. PD098059 treatment resulted in shortening of myotubes also in uninduced cultures, further suggesting a role of phospho-Erk1,2 in myoblast fusion. This observation fits with a previous study [21], which demonstrated that C2C12 cells failed to undergo appropriate cell-to-cell fusion to form multinucleated myotubes when Erk2 phosphorylation was inhibited by MAPK phosphatase-1 overexpression. Other studies have also shown that C2C12 myoblasts stably expressing small interfering RNA (siRNA) directed against Erk2 displayed severely impaired myoblast fusion [22]. Myoblast fusion is an early and essential step to the progression of the skeletal muscle differentiation program. The mechanisms and molecular actors implicated in myoblast fusion are fairly known. It thus remains to be understood the molecular determinants upregulated by the Erk1,2 MAPK pathway, through which Met hyperactivation promotes aberrant myotube fusion. Although a general role of the Erk1,2 MAPK pathway in the inhibition of muscle gene/protein expression (*i.e.*, downregulation of MyHC) is well established, its function in myoblast fusion may be, at first sight, contradictory. One hypothesis for this conflict may be that the balance between cell fusion and cell differentiation is required to dictate the biological outcome in response to MAPK activation during skeletal muscle differentiation.

Activated Met is able to induce cell cycle re-entry in quiescent SCs [23]. The Ras/RAF/MAPK pathway, strongly induced by activated Tpr–Met, is involved in driving the transcription of genes required for growth and cell division. By forcing the expression of a constitutively-active Met form in differentiated myotubes, we expected to push their nuclei back into the cell cycle and to revert their differentiation program. Our results demonstrate that terminally-differentiated myotubes mainly resist the mitogenic pressure of the Tpr–Met oncogene. The proliferation restricting mechanism must be very potent in post-mitotically-differentiated myotubes. Indeed, only viral oncogenes, such as SV40 large T antigen and adenovirus E1A, which are able to inactivate multiple checkpoint mechanisms [24], can reprogram post-mitotic nuclei in terminally-differentiated myotubes, inducing nuclear DNA synthesis without cell division [25,26,27]. In our work, we observed that DNA replication is induced by the Tpr–Met oncogene only in mononucleated cells upon DM shift. These cells could arise from fusion-defective differentiating cells during cell culture. It is notable that the BrdU+ myonuclei represent a low fraction of total nuclei (22%) in the Tpr–Met-expressing differentiated muscle cultures. In contrast, the majority of nuclei that remain aggregated in myosacs are prevented from re-entering the cell cycle. Constitutive activation of Met signaling in myotubes may contribute to mobilizing their nuclei and to stimulating their aggregation, as is observed in gigantic myosacs.

In conclusion, our data support the concept that multinucleated fused myotubes may be induced to disassemble their contractile apparatus by Tpr–Met. However, they are blocked from re-entering the cell cycle, even when forced by a powerful mitogenic oncogene, such as Tpr–Met.

## 4. Materials and Methods

### 4.1. Bitransgenic Mice

Mice harboring the MCK-tTA (skeletal muscle-specific) promoter construct were kindly donated by Dr. P. Plotz [14,28] and kept in a C57BL/6 background. MCK-tTA heterozygous mice were crossed with Tpr–Met–TRE–GFP heterozygous mice, and double-transgenic heterozygotes (Tpr–Met–TRE–GFP/MCK–tTA) were identified in the progeny by PCR analysis of tail genomic DNA, as previously described [10]. Doxycycline (DOX, Sigma, St. Louis, MO, USA) was diluted in 3% sucrose in water to a final concentration of 200 μg/mL and supplied as drinking water with changes every 2–3 days. *In vivo* GFP fluorescence was monitored by using a fluorescent light box, illuminated by blue light fiber optics and imaged by an Olympus Camedia camera. All animal procedures were approved by the Ethical Commission of the University of Turin (project identification: No. prot. 383 of 15 June 2005; approved on 10 June 2005), and by the Italian Ministry of Health, both of which accepted the use of mice for this study (A/R 0045 and A/R 0041).

### 4.2. Mouse Myogenic Culture Conditions and Differentiation Protocol

To isolate MSCs, we used the classical procedure, which involves the enzymatic dissociation of skeletal muscles with collagenase/dispase (Roche Applied Science, Indianapolis, IN, USA). Myogenic cultures were isolated from hindlimb muscles of 17-day-old double transgenic mice (MCKtTA/Tpr–Met–TRE–GFP), kept in DOX. After removal of skin, fat tissue and bones, hindlimb muscles were digested with 1 μg/μL collagenase/dispase in D-PBS supplemented with CaCl_2_ and MgCl_2_ (Sigma) for 40–50 min at 37 °C with shaking. During the proteolytic digestion, tissues were occasionally fragmented by repeated pipetting. The digestion was stopped by adding 1:3 fetal bovine serum in D-PBS. The debris was removed by filtration through a 70 µm sterile filter, and cells were collected by centrifugation. Cells were then resuspended in complete GM growth medium (F-10 HAM (Sigma)) containing 20% fetal bovine serum (FBS, Euroclone, Pero, Italy), 3% chicken embryo extract and 2.5 ng/mL bovine fibroblast growth factor (Peprotech, Rocky Hill, NJ, USA). Cells were pre-plated overnight to remove contaminating fibroblasts, and then non-adherent cells were plated in GM on collagen (0.1 mg/mL, Sigma) -coated plates. Single MSCs started to be visible after 2–3 days of culture. Cells were passaged every 3 days, when they were about 70% confluent, using EDTA 0.5 mM in PBS for detachment. The cellular population underwent a proliferation crisis after 2–3 weeks, from which proliferating MSCs arose. Under such conditions, the purity of MSCs exceeded 99%. Proliferating cells were cultured in GM growth medium on collagen-coated plates. They were cultured for 20–25 passages at maximum. To obtain differentiation into myotubes, cells were plated at subconfluence on gelatin-coated (0.5%, Sigma) plates, maintained in GM for 24 h and then shifted to DM differentiation medium (DMEM (Euroclone) containing 5% horse serum (Euroclone)). Incubation was performed at 37 °C in a 5% CO_2_–water-saturated atmosphere, and all media were supplemented with 2 mM l-glutamine, 100 U penicillin and 0.1 mg/mL streptomycin. DOX was added to the culture medium at the concentration of 2 μg/mL.

### 4.3. Inhibitors and Reagents

PD98059 (20 µM), Mg132 (5 µM) and PS-341 (6 nM) were added after 24 h from DM shift. All inhibitors were purchased from Calbiochem (Millipore, Billerica, MA, USA), except for PS-341, kindly donated by Millennium Pharmaceuticals (Cambridge, MA, USA). All reagents used were from Sigma.

### 4.4. Western Blot (WB) Analysis

Tissue samples and cell cultures were lysed at RT in lysis buffer at 4 °C in EB lysis buffer (20 mM Tris–HCl, pH 7.4; 160 mM NaCl; 10% glycerol; 5 mM EDTA, pH 8; 1% Triton-X-100; protease inhibitor cocktail; 1 mM sodium-orthovanadate). The protein concentration was determined by the BCA assay (Pierce, ThermoScientific, Rockford, IL, USA), and proteins were resolved in 10% SDS-PAGE gels (40 μg/lane) and transferred to Hybond-C Extra nitrocellulose membranes (Amersham Biosciences, Amersham, BKM, UK). The following antibodies were used: anti-human Met (Santa Cruz, #sc-10, Dallas, TX, USA); anti-phospho-Erk-1,2 MAPK (Sigma, #M8159); anti-total Erk2 (Santa Cruz, #sc-154); anti-α-tubulin (Sigma; #T5168); anti-MyHC MF20. HRP-conjugated goat anti-rabbit and rabbit anti-mouse antibodies were from Pierce. Immunoblots were developed with Super Signal West Pico Chemiluminescent Substrate (Pierce) and visualized on Amersham Hyperfilm. Each condition analyzed in WB was tested in 3 independent experiments. ImageJ (National Institutes of Health (NIH), Bethesda, MD, USA) was used to quantify blots.

### 4.5. Immunofluorescence (IF)

GFP fluorescence was monitored in living myotubes. For indirect immunofluorescence studies, myotubes differentiated on 24-well plates were fixed with 4% formaldehyde, permeabilized with 0.5% Triton X-100, incubated with primary antibody at the appropriate dilution (MF20 at 1:50; anti-α-tubulin at 1:1000 (Sigma; #T5168)), followed by incubation with Cy3-conjugated anti-mouse antibody (Sigma), then washed and briefly stained with DAPI (Sigma) to reveal nuclei. Samples were viewed under a fluorescence-equipped inverted DMRI (Digital Module R Inverted) microscope (Leica Microsystems, Wetzlar, Gießen, Germany). Pictures were taken with an Evolution VF color cool camera and Image-Pro software (Media Cybernetics, Rockville, MD, USA). Optical and IF experiments were analyzed in at least 3 independent experiments.

### 4.6. BrdU Incorporation Assay

To selectively detect BrdU incorporation in differentiated myotubes, MSCs were incubated for 2 days in DM; then, differentiated myotubes were cultured for a further 2 days in DM or in the presence of 10% FBS. Cells were incubated with 10 μM BrdU (Sigma) for a further 24 h. For the double-immunofluorescence assay, the cells were fixed with 4% formaldehyde, incubated with MF20 primary antibody followed by anti-mouse Alexa Fluor 488 conjugated antibody. After the secondary antibody incubation step, immune complexes were fixed with 95% ethanol, treated with 2 M HCl for 1 h and then incubated with BrdU-specific antibody-Alexa Fluor 546 conjugate plus 5 min with DAPI. The fraction of BrdU+ and DAPI+ nuclei was counted in double overlay pictures of red fluorescent BrdU and blue fluorescent DAPI. At least six fields of two different coverslips were reviewed in each experiment. All experiments were performed in duplicate in two independent experiments.

## References

[1] Olson E.N., Klein W.H. (1994). BHLH factors in muscle development: Dead lines and commitments, what to leave in and what to leave out. Genes Dev..

[2] Bischoff R. (1997). Chemotaxis of skeletal muscle satellite cells. Dev. Dyn..

[3] Cornelison D.D., Wold B.J. (1997). Single-cell analysis of regulatory gene expression in quiescent and activated mouse skeletal muscle satellite cells. Dev. Biol..

[4] Gal-Levi R., Leshem Y., Aoki S., Nakamura T., Halevy O. (1998). Hepatocyte growth factor plays a dual role in regulating skeletal muscle satellite cell proliferation and differentiation. Biochim. Biophys. Acta.

[5] Tatsumi R., Anderson J.E., Nevoret C.J., Halevy O., Allen R.E. (1998). HGF/SF is present in normal adult skeletal muscle and is capable of activating satellite cells. Dev. Biol..

[6] Rodgers J.T., King K.Y., Brett J.O., Cromie M.J., Charville G.W., Maguire K.K., Brunson C., Mastey N., Liu L., Tsai C.R. (2014). MTORC1 controls the adaptive transition of quiescent stem cells from G(0) to G(Alert). Nature.

[7] Yamada M., Tatsumi R., Yamanouchi K., Hosoyama T., Shiratsuchi S., Sato A., Mizunoya W., Ikeuchi Y., Furuse M., Allen R.E. (2010). High concentrations of HGF inhibit skeletal muscle satellite cell proliferation *in vitro* by inducing expression of myostatin: A possible mechanism for reestablishing satellite cell quiescence *in vivo*. Am. J. Physiol. Cell Physiol..

[8] Anastasi S., Giordano S., Sthandier O., Gambarotta G., Maione R., Comoglio P., Amati P. (1997). A natural hepatocyte growth factor/scatter factor autocrine loop in myoblast cells and the effect of the constitutive met kinase activation on myogenic differentiation. J. Cell Biol..

[9] Leshem Y., Spicer D.B., Gal-Levi R., Halevy O. (2000). Hepatocyte growth factor (HGF) inhibits skeletal muscle cell differentiation: A role for the BHLH protein twist and the Cdk inhibitor P27. J. Cell Physiol..

[10] Miller K.J., Thaloor D., Matteson S., Pavlath G.K. (2000). Hepatocyte growth factor affects satellite cell activation and differentiation in regenerating skeletal muscle. Am. J. Physiol. Cell Physiol..

[11] Dean M., Park M., Vande Woude G.F. (1987). Characterization of the rearranged *Tpr–Met* oncogene breakpoint. Mol. Cell Biol..

[12] Crepaldi T., Bersani F., Scuoppo C., Accornero P., Prunotto C., Taulli R., Forni P.E., Leo C., Chiarle R., Griffiths J. (2007). Conditional activation of MET in differentiated skeletal muscle induces atrophy. J. Biol. Chem..

[13] Gossen M., Bujard H. (1992). Tight control of gene expression in mammalian cells by tetracycline-responsive promoters. Proc. Natl. Acad. Sci. USA.

[14] Nagaraju K., Raben N., Loeffler L., Parker T., Rochon P.J., Lee E., Danning C., Wada R., Thompson C., Bahtiyar G. (2000). Conditional up-regulation of MHC class I in skeletal muscle leads to self-sustaining autoimmune myositis and myositis-specific autoantibodies. Proc. Natl. Acad. Sci. USA.

[15] Ponzetto C., Bardelli A., Zhen Z., Maina F., Dalla Z.P., Giordano S., Graziani A., Panayotou G., Comoglio P.M. (1994). A multifunctional docking site mediates signaling and transformation by the hepatocyte growth factor/scatter factor receptor family. Cell.

[16] Szewczyk N.J., Peterson B.K., Jacobson L.A. (2002). Activation of Ras and the mitogen-activated protein kinase pathway promotes protein degradation in muscle cells of caenorhabditis elegans. Mol. Cell Biol..

[17] Szewczyk N.J., Jacobson L.A. (2003). Activated EGL-15 FGF receptor promotes protein degradation in muscles of caenorhabditis elegans. EMBO J..

[18] Smith H.J., Tisdale M.J. (2003). Signal transduction pathways involved in proteolysis-inducing factor induced proteasome expression in murine myotubes. Br. J. Cancer.

[19] Palmer S., Groves N., Schindeler A., Yeoh T., Biben C., Wang C.C., Sparrow D.B., Barnett L., Jenkins N.A., Copeland N.G. (2001). The small muscle-specific protein csl modifies cell shape and promotes myocyte fusion in an insulin-like growth factor 1-dependent manner. J. Cell Biol..

[20] Yotov W.V., StArnaud R. (1996). Differential splicing-in of a proline-rich exon converts alpha NAC into a muscle-specific transcription factor. Genes Dev..

[21] Bennett A.M., Tonks N.K. (1997). Regulation of distinct stages of skeletal muscle differentiation by mitogen-activated protein kinases. Science.

[22] Li J., Johnson S.E. (2006). ERK2 is required for efficient terminal differentiation of skeletal myoblasts. Biochem. Biophs. Res. Commun..

[23] Allen R.E., Sheehan S.M., Taylor R.G., Kendall T.L., Rice G.M. (1995). Hepatocyte growth-factor activates quiescent skeletal-muscle satellite cells *in vitro*. J. Cell Physiol..

[24] Helt A.M., Galloway D.A. (2003). Mechanisms by which DNA tumor virus oncoproteins target the Rb family of pocket proteins. Carcinogenesis.

[25] Cardoso M.C., Leonhardt H., Nadal-Ginard B. (1993). Reversal of terminal differentiation and control of dna replication: Cyclin A and Cdk2 specifically localize at subnuclear sites of dna replication. Cell.

[26] Crescenzi M., Soddu S., Sacchi A., Tato F. (1995). Adenovirus infection induces reentry into the cell cycle of terminally differentiated skeletal muscle cells. Ann. N. Y. Acad. Sci..

[27] Endo T., Nadal-Ginard B. (1998). Reversal of myogenic terminal differentiation by SV40 large T antigen results in mitosis and apoptosis. J. Cell Sci..

[28] Ghersa P., Gobert R.P., Sattonnet-Roche P., Richards C.A., Merlo P.E., Hooft V.H. (1998). Highly controlled gene expression using combinations of a tissue-specific promoter, recombinant adenovirus and a tetracycline-regulatable transcription factor. Gene Ther..

